# Non-Thermal Plasma Sources Based on Cometary and Point-to-Ring Discharges

**DOI:** 10.3390/molecules27010238

**Published:** 2021-12-31

**Authors:** Josef Khun, Anna Machková, Petra Kašparová, Myron Klenivskyi, Eva Vaňková, Pavel Galář, Jaroslav Julák, Vladimír Scholtz

**Affiliations:** 1 Department of Physics and Measurements, University of Chemistry and Technology, 16628 Prague, Czech Republic; khunj@vscht.cz (J.K.); machkovn@vscht.cz (A.M.); kasparop@vscht.cz (P.K.); kvasnice@vscht.cz (E.V.); scholtzv@vscht.cz (V.S.); 2 Department of Thin Films and Nanostructures, Institute of Physics, Czech Academy of Sciences, 16200 Prague, Czech Republic; galarp@vscht.cz; 3 Institute of Immunology and Microbiology, First Faculty of Medicine, Charles University and General University Hospital, 12800 Prague, Czech Republic; jaroslav.julak@lf1.cuni.cz

**Keywords:** *Candida albicans*, corona discharge, *Pseudomonas aeruginosa*, microbicidal effect, *Staphylococcus aureus*, *Trichophyton interdigitale*

## Abstract

A non-thermal plasma (NTP) is a promising tool against the development of bacterial, viral, and fungal diseases. The recently revealed development of microbial resistance to traditional drugs has increased interest in the use of NTPs. We have studied and compared the physical and microbicidal properties of two types of NTP sources based on a cometary discharge in the point-to-point electrode configuration and a corona discharge in the point-to-ring electrode configuration. The electrical and emission properties of both discharges are reported. The microbicidal effect of NTP sources was tested on three strains of the bacterium *Staphylococcus aureus* (including the methicillin-resistant strain), the bacterium *Pseudomonas aeruginosa*, the yeast *Candida albicans*, and the micromycete *Trichophyton interdigitale*. In general, the cometary discharge is a less stable source of NTP and mostly forms smaller but more rapidly emerging inhibition zones on agar plates. Due to the point-to-ring electrode configuration, the second type of discharge has higher stability and provides larger affected but often not completely inhibited zones. However, after 60 min of exposure, the NTP sources based on the cometary and point-to-ring discharges showed a similar microbicidal effect for bacteria and an individual effect for microscopic fungi.

## 1. Introduction

Recently, resistance to commonly used antibiotics has been demonstrated in most clinically relevant pathogens [1,2,3,4,5,6]. The ability of microorganisms to develop defense mechanisms against traditional drug therapy has led to the necessity to search for new possible treatment methods. A non-thermal plasma (NTP) is a promising tool for the treatment microbial infections due to its special mechanism of action, which is based, for example, on damage to the microbial membrane, as described in a comprehensive review of Liao et al. [7] or Scholtz et al. [8].

NTP is usually generated by the electric discharges of various types, as summarized in different studies [9,10,11,12,13]. The most common electric discharges used as sources of NTP are various types of DC discharges in the air [14,15], atmospheric pressure plasma jets (including plasma needle, plasma torch, and plasma pen) [16,17], dielectric barrier discharges [18], gliding arc discharges [19], microwave discharges [20], and others.

In works [21,22], we reported a new type of DC discharge formed in the air with some interesting and promising characteristics. We named it a cometary discharge because of its specific appearance resembling a comet’s tail, which is similar to an atmospheric pressure plasma jet. The most attractive feature of the cometary discharge is that it creates a flow of plasma species propagating from the tail. The flow of plasma species makes it possible to treat indirectly various surfaces in the air as a plasma jet but without the external gas supply.

**Figure 1:**
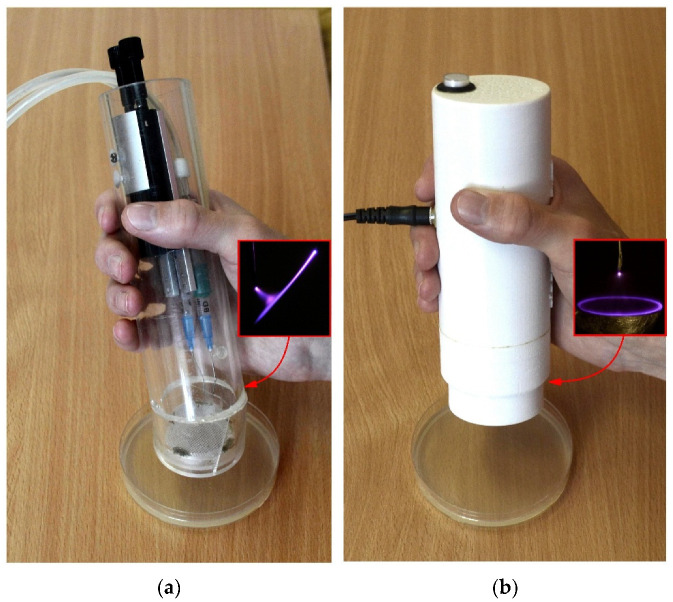
The developed sources of NTP: (**a**) source of NTP based on the cometary discharge; (**b**) source of NTP based on the point-to-ring discharge.

Atmospheric pressure plasma jets require the injection of working gas into the discharge area. This makes such an NTP source more expensive and less portable. In contrast to atmospheric pressure plasma jets, the cometary discharge forms a plasma jet in the air without injecting gas into the discharge region. Moreover, the cometary discharge does not require an expensive pulsed, AC, or RF high-voltage power supply. Therefore, an NTP source based on the cometary discharge is a cheap, simple, and portable device (Figure 1a), which requires only two needle electrodes and a high-voltage DC power supply. This type of discharge has been used in a number of different applications: for human skin disinfection, healing of dermatomycosis in animal models and human patients, for the treatment of human onychomycosis [21,22,23,24,25,26,27,28,29]. It has been shown to be a source of NTP with a well-pronounced microbicidal and even antibiofilm effect [25,29]. However, the cometary discharge requires solving the problem of its stabilization for a more reliable operation. Unfortunately, this issue has not received due attention.

Later, our group adopted a DC corona discharge in the point-to-ring electrode configuration for treating various surfaces in the air. In contrast to the cometary discharge, the point-to-ring discharge does not form a plasma jet but tangibly blows out plasma species through the ring electrode without pumping any feed gas. The NTP source based on the point-to-ring discharge is also a cheap, simple, and portable device (Figure 1b), which, in principle, requires only two electrodes and an inexpensive low-power DC high-voltage supply. For ease of use, the source of NTP is made in a compact plastic case printed on a 3D printer and is a ready-made device. The NTP source based on the point-to-ring discharge has already been successfully used for the inactivation of fungi [30,31].

The NTP sources based on the cometary and point-to-ring discharges are actively used in our practice for a variety of applications. We take advantage of their simplicity, low cost, portability, and the pronounced property of blowing out plasma species. However, we found it necessary to obtain systematic data on the microbicidal efficiency of the cometary and point-to-ring discharges to enable the selection of the appropriate NTP source for a particular application.

This work aims at studying and comparing the electrical, emission, and microbicidal properties of the NTP sources based on the cometary and point-to-ring discharges as well as presenting their features and prospects.

## 2. Results

### 2.1. Electrical Properties of the Cometary and Point-to-Ring Discharges

**Figure 2:**
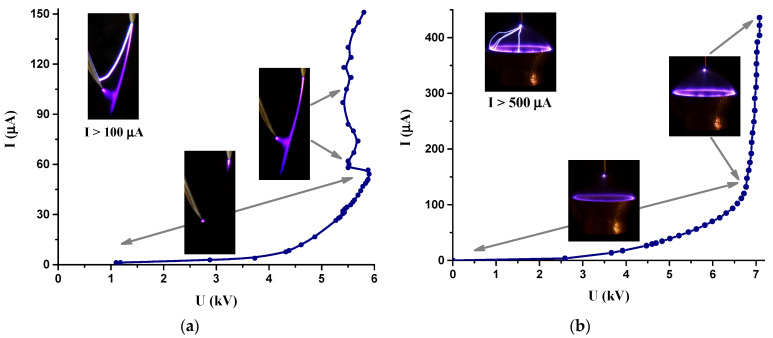
The volt–ampere characteristics and relevant images of the cometary discharge (**a**) and the point-to-ring discharge (**b**).

The volt–ampere (VA) characteristic of the cometary discharge in the point-to-point electrode system is shown in Figure 2a. In the initial part of the characteristic, corresponding to discharge currents of less than 60 μA, a bipolar corona discharge was observed. The cometary discharge took place in the range of the discharge currents of 60 to 150 μA. However, the discharge in this range was rather unstable. At discharge currents of 60 to 80 μA, the cometary discharge did not last long enough and disappeared, switching to the bipolar corona discharge mode. At higher currents, the cometary discharge was accompanied by random single sparks, the number of which increased with increasing current. At discharge currents higher than 100 μA, the cometary discharge could spontaneously switch to the transient spark regime, and the higher the discharge current, the higher the transition probability. When the discharge current exceeded 150 μA, only a transient spark discharge was observed.

As can be seen in Figure 2a, the part of the VA characteristic that corresponds to the cometary discharge mode is not monotonic, indicating poor stability of the cometary discharge. According to our observations, the cometary discharge appeared to be the most stable in the range of discharge currents of 80 to 100 μA. However, even under these conditions, the discharge was quite unstable and behaved as an unsteady flame changing its shape. The dimensions of the cometary discharge and the discharge current continuously fluctuated, increasing and decreasing in magnitude. It should be mentioned that no special measures were taken to stabilize the cometary discharge.

During the study of the microbicidal effect of the cometary discharge, the discharge current was approximately 90 μA, and the discharge voltage was 5.5 kV. The electric power delivered to the cometary discharge plasma was approximately 0.5 W.

Figure 2b shows a VA characteristic of a discharge in the point-to-ring electrode system. The characteristic can be divided into two parts corresponding to different discharge regimes. The first part up to the discharge voltage of approximately 6.5 kV corresponds to a bipolar corona discharge, while the second part with higher discharge voltages corresponds to a glow discharge. A further increase in the discharge voltage, when the discharge current exceeded 500 μA, resulted in the transition of the discharge in the point-to-ring electrode system to the spark regime. As can be seen, the VA characteristic of the discharge in the point-to-ring electrode system has a rather smooth and monotonic course. This indicates a high stability of both the bipolar corona and glow discharge regimes.

The microbicidal effect of a discharge in the point-to-ring electrode system was studied at the discharge voltage of 6.7 kV, which corresponded to the discharge current of 150 μA. The power delivered to the discharge plasma was 1 W (twice as high as in the case of the cometary discharge). Under these conditions, the discharge in the point-to-ring electrode system operated at the border between the bipolar corona and glow regimes. Nevertheless, we still refer to this discharge as a corona, since the glow discharge is not fully developed under these conditions.

### 2.2. Emission Properties of the Cometary and Point-to-Ring Discharges

Despite the differences in the electrical parameters of the cometary and point-to-ring discharges, the spectral compositions of their radiation did not differ significantly, indicating a similar composition of the cometary and point-to-ring discharge plasmas. Further, we present a general description of their emission spectra.

**Figure 3:**
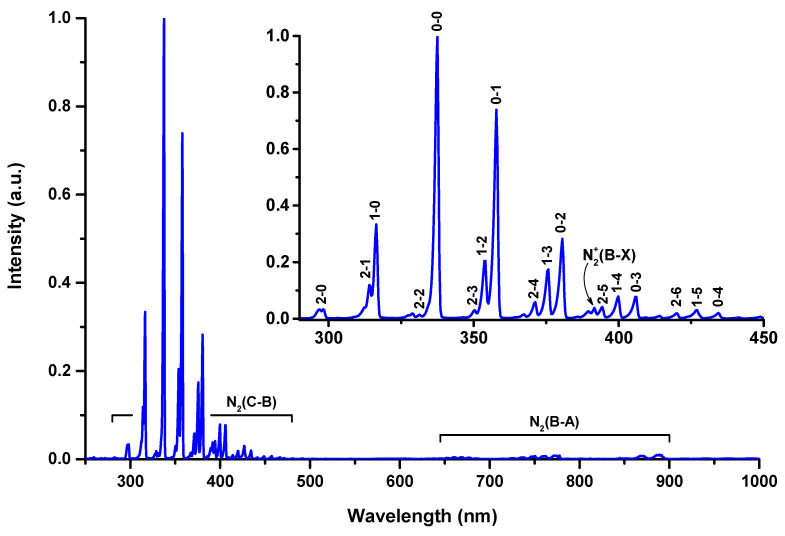
Typical time-integrated emission spectrum of the cometary/point-to-ring discharge. The inset shows the short-wavelength region of the emission spectrum.

Figure 3 shows a typical time-integrated emission spectrum of the cometary and point-to-ring discharges in the spectral range from 250 to 1000 nm. The inset in Figure 3 shows the short-wavelength region of the emission spectrum with the peaks interpreted. The pairs of numbers near the peaks indicate vibrational quantum numbers corresponding to the transitions of the second positive system. The peaks were identified by comparing the measured and calculated wavelengths. The wavelengths of the electronic–vibrational transitions were calculated using vibrational constants of the nitrogen molecule [32,33].

As can be seen in Figure 3, the emission spectrum is dominated by the second positive system of the nitrogen molecule, N2(C Πu3)→N2(B Πg3). In the long-wavelength region of the spectrum, the first positive system of the nitrogen molecule, N2(B Πg3)→N2(A Σu+3), with significantly lower intensity was observed. The emission of the first negative system of the ionized nitrogen molecule, N2+(B Σu+2)→N2+(X Σg+2), was also registered, but its intensity did not exceed a few percent relative to the main peak in the emission spectrum, which is the N2(C Πu3[υ=0])→N2(B Πg3[υ=0]) electronic–vibrational transition (λ = 337.1 nm).

Spectral lines of oxygen atoms, corresponding to 3p P5− 3s S05 and 3p P3− 3s S03 resonance transitions, were also registered in the spectrum (λ = 777.4 and 844.6 nm, respectively), but their intensities were about 1%. The emission of hydroxyl radical as well as spectral lines of hydrogen, which are often observed in an air plasma, were not detected. Furthermore, no spectral lines of atomic nitrogen were observed in the emission spectra of the discharges. That was due to the relatively low specific power introduced into the discharge plasmas.

**Figure 4:**
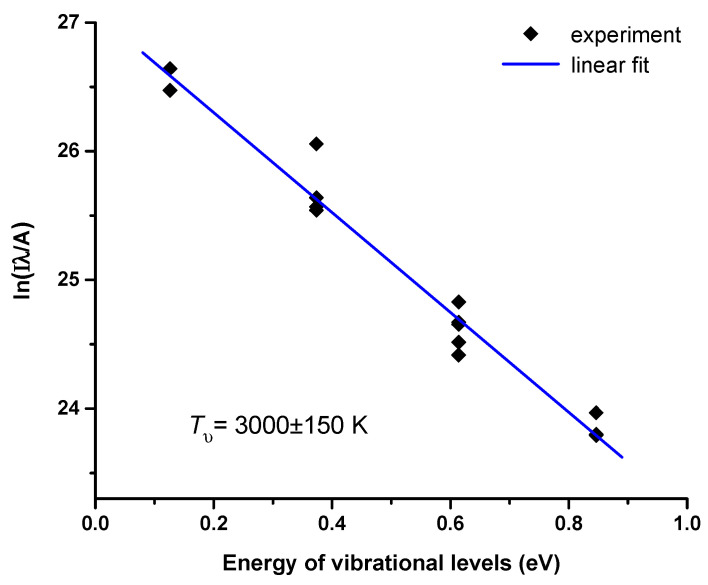
Boltzmann plot estimating the vibrational temperature of the nitrogen molecules.

The spectral intensity distribution of the second positive system indicates a relatively high vibrational temperature of the nitrogen molecules. To estimate it, we used the Boltzmann plot method [34,35]. Figure 4 shows a Boltzmann plot of the distribution of the N2(C Πu3) nitrogen molecules over the first vibrational energy levels. The Boltzmann plot was obtained by integrating the emission intensity of the individual vibrational bands in the second positive system. The probabilities of the electronic–vibrational transitions required for the Boltzmann plot were taken from the paper [36]. The slope of the linear fit to the Boltzmann plot gave the vibrational temperature of *T*_υ_ ≈ 3000 K. Note that the partial overlap of the vibrational bands makes it difficult to estimate the vibrational temperature with high accuracy using the Boltzmann plot method.

The composition of the emission spectrum indicates that the generated plasma must contain an abundance of N2(A Σu+3) metastable nitrogen molecules, which is consistent with other works [37,38,39,40]. This is because this metastable state is the first excited electronic state of the nitrogen molecule and, on the other hand, it is a long-lived state with a radiative lifetime of 2 s [36]. The metastable nitrogen molecules are produced by direct electron impact excitation from the ground state [41]:

(1) N2(X Σg+1)+e→N2(A Σu+3)+e′

and via radiative decay of excited nitrogen molecules

(2) N2(C Πu3)→N2(B Πg3)+hv,

(3) N2(B Πg3)→N2(A Σu+3)+hv.

As one can see, the radiative decay of the N2(C Πu3) nitrogen molecules populates the N2(B Πg3) nitrogen molecules. Moreover, the effective cross-section for the electron impact excitation of the N2(B Πg3) nitrogen molecule is comparable to that of the N2(C Πu3) nitrogen molecule [42]. Therefore, the fact that the intensity of the first positive system is significantly lower than the intensity of the second positive system can be explained by a collisional quenching of the N2(B Πg3) nitrogen molecules by air species. The radiative lifetime of the N2(B Πg3) nitrogen molecule is 11 μs, and the radiative lifetime of the N2(C Πu3) nitrogen molecule is 37 ns [36]. Estimates show that the N2(C Πu3) nitrogen molecule experiences single collisions during the radiative lifetime, while the N2(B Πg3) nitrogen molecule undergoes several hundred collisions by air species before emitting a photon.

Metastable nitrogen molecules are known to play an important role in plasma kinetics and are responsible for some key plasma–chemical processes [43,44]. Furthermore, having a long radiative lifetime and excitation energy of 6.17 eV [32], metastable nitrogen molecules may well initiate some plasma–chemical processes outside the discharge region. For example, it is known that the metastable nitrogen molecules can cause the generation of ozone [37,44,45,46]. The excitation energy of the metastable nitrogen molecule is high enough to dissociate the oxygen molecule (5.17 eV) and form oxygen atoms

(4) N2(A Σu+3)+O2→N2(X Σg+1)+2O.

Then, the produced oxygen atoms form ozone in collisions with oxygen molecules and a third partner

(5) $$O+O_{2}+M\to O_{3}+M.$$

The third collision partner, *M*, takes the excess energy to stabilize the ozone molecule. As a rule, nitrogen molecules in the ground state act as the third collision partner due to their abundance in the air. It is known that ozone is effectively produced in the post-discharge time [46] and thus can be formed outside the discharge region. It should be mentioned that the generation of ozone took place in our discharge plasmas. The characteristic smell of ozone was perceptible both in the cometary and point-to-ring discharges.

In addition, the metastable nitrogen molecule can dissociate a water molecule (5.15 eV) and form a hydroxyl radical [47]

(6) N2(A Σu+3)+H2O→N2(X Σg+1)+OH+H.

Thus, the energy stored in the metastable nitrogen molecules can be transported outside the discharge region and released there, leading to the formation of active species.

### 2.3. Microbicidal Properties of the Cometary and Point-to-Ring Discharges

The microbicidal effects of cometary and point-to-ring discharges on the individual microorganisms are summarized in Table 1 and Table 2. Table 1 presents areas of incomplete inhibition where the population of survived microorganisms was noticeably reduced, and Table 2 comprises areas of complete inhibition, containing no surviving microorganisms.

**Figure 5:**
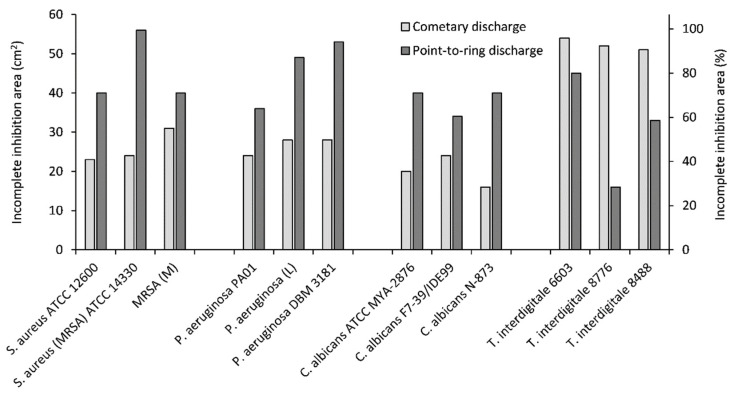
Areas of incomplete inhibition after exposure of individual microorganisms to the cometary/point-to-ring discharge for 60 min.

**Figure 6:**
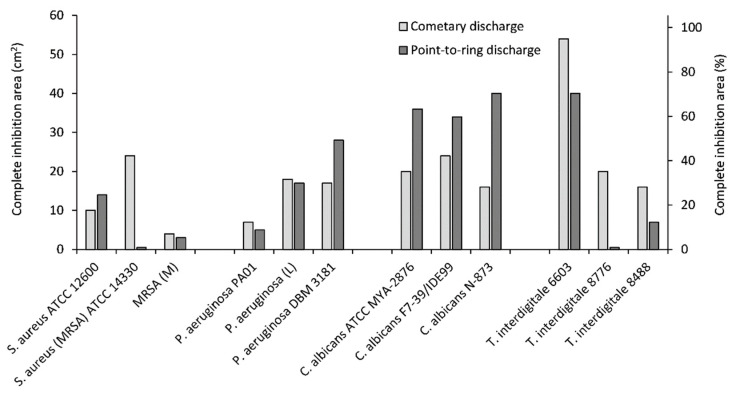
Areas of complete inhibition after exposure of individual microorganisms to the cometary/point-to-ring discharge for 60 min.

Areas of incomplete inhibition were larger than areas of complete inhibition for the same strains, with both these areas regularly increasing with exposure time. For a better comparison, the areas of incomplete and complete inhibition of the tested microorganisms after the maximum exposure time of 60 min are shown in Figure 5 and Figure 6, respectively.

As can be seen in Figure 5, the point-to-ring discharge showed better results in terms of incomplete inhibition area for all tested microorganisms except *T. interdigitale* strains. Similar results were observed at most other exposure times except for 5 min (see Table 1). *T. interdigitale* generally showed the highest sensitivity to the cometary discharge exposure, with incomplete inhibition of more than 90% in all tested strains after 60 min of exposure and complete inhibition of *T. interdigitale* 6603, reaching 96% under the same conditions.

Figure 6 demonstrates that the cometary discharge had better results in terms of complete inhibition area for most tested microorganisms except for *C. albicans* strains, *S. aureus* ATCC 12600, and *P. aeruginosa* DBM 3181. For these microorganisms, the point-to-ring discharge had advantage over the cometary discharge in the view of both complete and incomplete inhibition areas. For instance, the point-to-ring discharge resulted in more than 60% complete inhibition area after 60 min of exposure for all tested strains of *C. albicans* yeast. It is worth noting that the cometary discharge was somewhat more effective for the treatment of *T. interdigitale* strains in terms of not only complete inhibition area but also of incomplete one.

From the above, one can conclude that in terms of complete inhibition zones, the cometary discharge was usually more effective, and, conversely, the point-to-ring discharge showed better results in the view of incomplete inhibition zones. When using the cometary discharge, the areas of incomplete inhibition zones for a given microorganism were very similar, and when using the point-to-ring discharge, they were very dependent on the individual strain of a given microorganism. On a detailed comparison, it is noticeable that different strains of the same microorganism show different sensitivities to the same discharge.

Comparing the areas of incomplete inhibition zones of *S. aureus*, one can see (Table 1) that at the shortest exposure time, the cometary discharge had a higher effect, but at longer exposure time, the point-to-ring discharge prevailed (with more than 50% incomplete inhibition after 30 min for all strains tested). The same trend was also observed for *P. aeruginosa*. In the case of the incomplete inhibition zone of *C. albicans*, the effect of the point-to-ring discharge was already higher, starting with the shortest exposure time.

## 3. Discussion

The microbicidal activity of different NTP sources usually varies considerably. This fact was comprehensively summarized, among others, in our recent review [8], where we described the different ways of generating NTP and the variability of its microbicidal effect on the most life-threatening microorganisms. It is obvious that this activity depends mainly on the NTP source used and that the difference in the microbicidal effect is often observed even between very similar NTP sources. Overall, in terms of treatment time, plasma jets are the most efficient sources of NTP, as complete suppression of microbial growth was typically achieved in several minutes in a large number of studies [8,48,49,50,51]. Although it may seem that further development of NTP sources is unnecessary, as there are already a large number of prototypes, it should be noted that specific applications require different suitable devices. As the main one, the mentioned plasma jets are able to treat only a relatively small area of the exposed surface. On the contrary, corona discharges, also studied in this work, are used for the treatment of much larger areas. Their possible application is the sterilization of medical instruments, food packaging, etc., and their microbicidal effect is usually achieved from half an hour to several hours of exposure [25,52,53,54].

Our research group has focused especially on the treatment of different microorganisms using the cometary discharge. This discharge was developed about ten years ago [21,22], and its capabilities and usefulness have been demonstrated in many works dealing with inhibition of bacteria, fungi including yeasts, and prions [55]. However, the use of the cometary discharge is limited by the instability outside the narrowly defined range of its operating parameters; therefore, it was gradually replaced by the discharge in the point-to-ring electrode configuration, which was expected to have an efficiency similar to that of the cometary discharge. The physical parameters and microbicidal action of both the cometary and point-to-ring discharge were evaluated and compared in this study.

The results showed that the point-to-ring discharge is better defined and is much more stable than the cometary one. In addition, the cometary discharge was better in the view of complete inhibition, and the point-to-ring discharge affected a larger area but with a less pronounced microbicidal effect. To find out the reason for the different action of the cometary and point-to-ring discharges, we studied their emission spectra. However, the spectral characteristics of the discharges did not differ qualitatively, indicating a similar composition of both plasmas.

It is generally accepted that the microbiocidal activity of plasma is mediated by reactive oxygen and reactive nitrogen species produced in the plasma. The kinetics of plasma–chemical reactions is rather complex. The microbicidal action of these reactive particles was studied in different works (see, e.g. [56,57,58,59]). The role of UV radiation is also widely discussed. Some studies show that UV radiation of plasma is of importance [60,61], but some works impugn it [62,63]. This ambiguity is due to the different types of discharges used. However, according to work [64], UV radiation of low-pressure discharge plasma usually plays a significant role, while the microbicidal action of UV radiation from atmospheric-pressure cold plasma is negligible. This conclusion is consistent with other works [63,65,66,67,68]. Thus, it can be assumed that the contribution of UV radiation to the microbicidal action of the cometary and point-to-ring discharges is also insignificant.

Similar composition of plasmas produced by the cometary and point-to-ring discharges indicates that the observed differences in the microbicidal action of the discharges must consist in the spatial distribution of plasma species falling onto the treated surface. Thus, the cometary discharge has a higher density of plasma species in the middle, while the point-to-ring one has a more distributed density of plasma species over the whole surface.

However, detailed calculations using the data for the maximum exposure time of 60 min showed that in total, over all microorganisms, the difference in the areas of inhibition by the cometary and point-to-ring discharges did not exceed 15%. Thus, at long exposure times, both discharges have approximately the same microbicidal efficiency for the inactivation of various microorganisms.

## 4. Materials and Methods

### 4.1. Non-Thermal Plasma Generation

**Figure 7:**
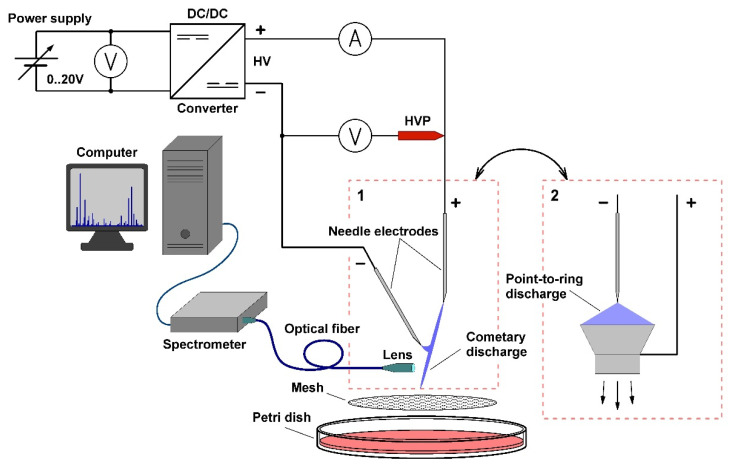
Experimental setup: 1—point-to-point electrode configuration for the cometary discharge, 2—point-to-ring electrode configuration for the corona discharge.

The cometary and point-to-ring discharges were generated using two-electrode configurations. The experimental setup is illustrated in Figure 7. The cometary discharge used was previously described in detail in works [21,22,27]. In short, it was formed between two needle electrodes (Medoject 0.6 mm × 25 mm intramuscular injection needles). The electrode system of the cometary discharge is shown as configuration 1 in Figure 7. The needles were positioned at an angle of 20–30° to each other, the horizontal and vertical gap between the tips was 2 and 4.5 mm, respectively. In practical terms, the dominant part of the discharge is the plasma jet, which burns from the upper positive electrode and blows reactive particles from the discharge onto the exposed surface.

The point-to-ring discharge was generated using a needle-ring configuration of electrodes (configuration 2 in Figure 7). The ring electrode was conical in shape and was made of brass. The top of the electrode was approximately 11 mm in diameter and was 3.3 mm below the tip of the needle electrode (a Medoject 0.6 mm × 25 mm intramuscular injection needle). The ring electrode was connected to the positive terminal of a high-voltage source, while the needle electrode was connected to the negative one. In the vicinity of the tip, the negative corona discharge is formed, and the positive one burns on the edge of the ring. Thus, we obtain a bipolar corona discharge, where reactive species created in the plasma are entrained by ions accelerated in the electric field between the electrodes. While most of the charged particles from the negative corona are obviously attracted to the ring electrode, neutral reactive particles are blown through the ring electrode out of the discharge onto the surface to be treated.

The electrode systems were fed by a high-voltage power supply composed of a DC voltage source varied in the range of 0–20 V and a step-up high-voltage (up to 20 kV) DC/DC converter with the galvanically separated primary and secondary circuits. No additional ballast resistance was employed, since the used DC/DC converter had a high output impedance.

To test the microbicidal action of the used discharges, we exposed various microorganisms (for details, see Section 4.4 and Section 4.5) to the generated plasmas. During plasma exposure, inoculated Petri dishes were 2.5 cm below the electrode systems. The exposure time was controlled by a timer. To distribute the flow of plasma species blown out by the discharges over the area of a Petri dish, we placed a metal mesh under each discharge. The meshes were neither grounded nor energized. The beneficial effect of a metal mesh was reported in [23,27].

### 4.2. Electrical Properties of the Discharges

To compare cometary and point-to-ring discharges, we controlled their electrical parameters. The discharge current was measured directly with a Metex M-3890DT digital multimeter (Metex Instruments, Toronto, Canada), and the discharge voltage was monitored with a Metex M-3800 digital multimeter (Metex Instruments, Toronto, ON, Canada) with a 1000:1 Pintek HVP-40 high-voltage probe (Pintek Electronics Co., Ltd., New Taipei, Taiwan).

### 4.3. Emission Properties of the Discharges

In addition to the electrical parameters, we analyzed the compositions of the plasmas generated by the cometary and point-to-ring discharges by studying and comparing their emission spectra. The emission spectra were recorded using a Shamrock-300i Czerny-Turner spectrograph (Andor, Oxford Instruments, Abingdon, UK) equipped with a Newton 971 EMCCD camera (Andor, Oxford Instruments, Abingdon, UK). The discharge radiation was fed into the spectrometer via an optical fiber. The emission spectra were recorded over the range of 250–1000 nm with a resolution of 1 nm and were corrected for the spectral sensitivity of the optical system.

### 4.4. Microbial Strains

To study the microbicidal effects of the cometary and point-to-ring discharges, we used some clinically important pathogens of Gram-positive and Gram-negative bacteria and fungi. Three strains of *Staphylococcus aureus* were chosen as representatives of Gram-positive bacteria: collection strains *S. aureus* ATCC 12600 (type strain) and methicillin-resistant (MRSA) *S. aureus* ATCC 14330; a clinical isolate of MRSA (M), provided by Motol University Hospital, Prague, Czech Republic.

Gram-negative bacteria were represented by the following strains: the reference strain *Pseudomonas aeruginosa* PAO1; a clinical isolate *P. aeruginosa* DBM 3181, kindly provided by the Department of Biochemistry and Microbiology (DBM), University of Chemistry and Technology, Prague; a clinical isolate of *P. aeruginosa* (L.) originated from Liberec Regional Hospital, Liberec, Czech Republic.

Yeast pathogens were as follows: *C. albicans* ATCC MYA-2876 (SC 5314, reference strain, clinical specimen from human); a clinical isolate *C. albicans* F7-39/IDE99, received from Palacký University Olomouc from a patient with oropharyngeal candidiasis; *C. albicans* N-873, obtained from the Department of Medical Microbiology, 2nd Faculty of Medicine, Charles University in Prague and Motol University Hospital, from a patient with vulvovaginal candidiasis.

Fungal pathogens were represented by three strains of *Trichophyton interdigitale* clinical isolates, kindly provided by Laboratory of Clinical Mycology, Public Health Institute in Ostrava: *T. interdigitale* 6603, isolated from nails; *T. interdigitale* 8776 from the lesion on the forearm skin; *T. interdigitale* 8488 from the neck skin lesion.

The Gram-positive bacteria were cultivated on Tryptone Soya Agar (Oxoid, Basingstoke, UK) for 24 h at 37 °C. The Gram-negative ones were cultivated on Luria–Bertanni Agar (Oxoid, Basingstoke, UK) for 24 h at 37 °C. The fungi were cultivated on Sabouraud Agar (Oxoid, Basingstoke, UK) for 48 h at 37 °C in the case of *C. albicans* and 6 days at 25 °C in the case of *T. interdigitale*. Stock suspensions of all microorganisms were stored at −70 °C in 50% glycerol before use.

### 4.5. Evaluation of Microbicidal Properties

The microbicidal efficiency of the NTP sources was evaluated by measuring the areas of inhibition zones on agar plates. The inoculum was prepared from the glycerol stocks suspensions of studied strains by dilution in saline and adjusted to the optical density of OD_600nm_ = 0.022 ± 0.002, corresponding to the concentration of bacteria of 3 × 10^7^ CFU/mL (colony-forming units per milliliter). The concentration of fungi was adjusted to 2.5 × 10^5^ CFU/mL. An aliquot of 600 µL of the inoculum was evenly distributed over the entire agar surface. Then, the inoculated plates were exposed to the cometary or point-to-ring discharge for 0, 5, 15, 30, and 60 min and subsequently cultivated under the conditions described above (see Section 4.4). To reveal a possible thermal effect of plasma on microorganisms, we measured the temperature of the agar surface using a FLIR E4 Thermal Imaging Camera. However, even during 60 min of exposure, the temperature of the agar surface did not deviate by more than 1–2 °C, which indicates that the plasma has no thermal effect.

**Figure 8:**
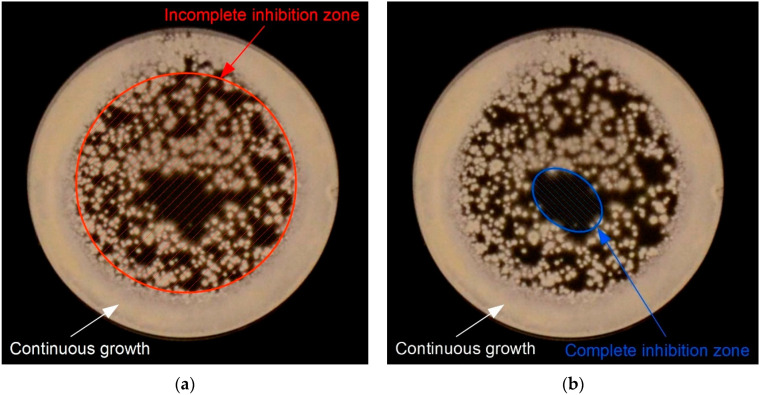
An example of the determination of inhibition zones for the evaluation of the microbicidal properties: (**a**) determination of an incomplete inhibition zone; (**b**) determination of a complete inhibition zone.

The microbicidal efficiency was determined as the area of inhibition zones on exposed agar plates after cultivation. A typical appearance of the exposed samples is an area with no microbial growth in the middle surrounded by an area of low microbial colony density. This area with a noticeably reduced population of microorganisms was considered as an incomplete inhibition zone, and an area with no microbial growth was considered as a complete inhibition zone (see Figure 8 for details). The experiments were carried out in triplicate for each microbial strain. The evaluated areas of inhibition zones were averaged and standard deviations were calculated.

## 5. Conclusions

The physical and microbicidal properties of NTP sources based on the cometary discharge and point-to-ring discharge were compared. Despite some differences in the electrical parameters, optical emission spectroscopy revealed no significant differences in the emission spectra of the cometary and point-to-ring discharges, indicating a similar composition of both plasmas.

As far as the microbicidal properties of the discharges are concerned, the cometary discharge was usually better in terms of complete inhibition area, while the point-to-ring discharge generally affected a larger area but with a less pronounced microbicidal effect. The cometary discharge is best suited for more localized treatment, and the point-to-ring discharge is better suited for treating a wider area. The choice of a suitable source of NTP depends on the desired medical needs and, in combination with conventional drug therapy, can potentially improve the treatment of various pathogens.

Overall, the NTP source based on the point-to-ring discharge met our expectations. At long exposure times, it has a microbicidal effect similar to that of the cometary one but is more stable and reliable in operation. The working conditions of the point-to-ring discharge are as follows: the discharge voltage is 6.7 kV, the discharge current is 150 μA, and the interelectrode gap is 3.3 mm.

## Figures and Tables

**Table 1:** Areas of incomplete inhibition (in cm^2^) after exposure of individual microorganisms to the cometary/point-to-ring discharge.

Discharge	Cometary						Point-to-Ring				
**Exposure Time (min)**	**0**	**5**	**15**	**30**	**60**		**0**	**5**	**15**	**30**	**60**
*S. aureus* ATCC 12600	0	12	19	20	23		0	0	31	39	40
*S. aureus* (MRSA) ATCC 14330	0	8	19	21	24		0	0	45	55	56
MRSA (M)	0	20	21	24	31		0	0	24	35	40
											
*P. aeruginosa* PA01	0	4	16	18	24		0	0	0	16	36
*P. aeruginosa* (L)	0	10	18	25	28		0	0	21	45	49
*P. aeruginosa* DBM 3181	0	13	20	24	28		0	0	46	53	53
											
*C. albicans* ATCC MYA-2876	0	4	5	16	20		0	31	44	48	40
*C. albicans* F7-39/IDE99	0	4	12	22	24		0	17	26	32	34
*C. albicans* N-873	0	0	13	15	16		0	0	0	15	40
											
*T. interdigitale* 6603	0	27	41	51	54		0	0	14	37	45
*T. interdigitale* 8776	0	23	39	50	52		0	0	6	18	16
*T. interdigitale* 8488	0	30	43	51	51		0	0	7	26	33

**Table 2:** Areas of complete inhibition (in cm^2^) after exposure of individual microorganisms to the cometary/point-to-ring discharge.

Discharge	Cometary						Point-to-Ring				
Exposure Time (min)	**0**	**5**	**15**	**30**	**60**		**0**	**5**	**15**	**30**	**60**
*S. aureus* ATCC 12600	0	0	1	3	10		0	0	0	6	14
*S. aureus* (MRSA) ATCC 14330	0	0	0	18	24		0	0	0	0	0
MRSA (M)	0	0	0	3	4		0	0	0	3	3
											
*P. aeruginosa* PA01	0	0	1	2	7		0	0	0	0	5
*P. aeruginosa* (L)	0	0	3	12	18		0	0	0	3	17
*P. aeruginosa* DBM 3181	0	0	1	4	17		0	0	0	1	28
											
*C. albicans* ATCC MYA-2876	0	4	5	13	20		0	6	14	24	36
*C. albicans* F7-39/IDE99	0	4	12	22	24		0	15	26	32	34
*C. albicans* N-873	0	0	5	10	16		0	0	0	15	40
											
*T. interdigitale* 6603	0	16	39	51	54		0	0	0	16	40
*T. interdigitale* 8776	0	0	1	13	20		0	0	0	0	0
*T. interdigitale* 8488	0	1	1	9	16		0	0	0	0	7

## Data Availability

All data are available upon request from the authors.

## Sample Availability

Microbial samples used are available from the authors.
